# Gamification for health and wellbeing: A systematic review of the literature

**DOI:** 10.1016/j.invent.2016.10.002

**Published:** 2016-11-02

**Authors:** Daniel Johnson, Sebastian Deterding, Kerri-Ann Kuhn, Aleksandra Staneva, Stoyan Stoyanov, Leanne Hides

**Affiliations:** a Queensland University of Technology (QUT), GPO Box 2434, Brisbane, QLD 4001, Australia; b Digital Creativity Labs, University of York, York YO10 5GE, United Kingdom

**Keywords:** Gamification, Health, Wellbeing, Systematic review

## Abstract

**Background:**

Compared to traditional persuasive technology and health games, gamification is posited to offer several advantages for motivating behaviour change for health and well-being, and increasingly used. Yet little is known about its effectiveness.

**Aims:**

We aimed to assess the amount and quality of empirical support for the advantages and effectiveness of gamification applied to health and well-being.

**Methods:**

We identified seven potential advantages of gamification from existing research and conducted a systematic literature review of empirical studies on gamification for health and well-being, assessing quality of evidence, effect type, and application domain.

**Results:**

We identified 19 papers that report empirical evidence on the effect of gamification on health and well-being. 59% reported positive, 41% mixed effects, with mostly moderate or lower quality of evidence provided. Results were clear for health-related behaviours, but mixed for cognitive outcomes.

**Conclusions:**

The current state of evidence supports that gamification can have a positive impact in health and wellbeing, particularly for health behaviours. However several studies report mixed or neutral effect. Findings need to be interpreted with caution due to the relatively small number of studies and methodological limitations of many studies (e.g., a lack of comparison of gamified interventions to non-gamified versions of the intervention).

## Highlights

• A systematic review is conducted to assess the empirical effectiveness of gamification in the health and wellbeing domain.
• Twenty-one papers are identified that report empirical evidence on the effectiveness of gamification in health and wellbeing.
• Overall the evidence suggests gamification can have a positive impact for health and wellbeing related interventions.
• The evidence is strongest for the use of gamification to target behavioural outcomes, particularly physical activity.
• Further research that isolates the impacts of gamification is needed.

## 1 Introduction

### 1.1 Background

The major health challenges facing the world today are shifting from traditional, pre-modern risks like malnutrition, poor water quality and indoor air pollution to challenges generated by the modern world itself. Today, the leading global risks for mortality and chronic diseases – high blood pressure, tobacco use, high blood glucose, physical inactivity, obesity, high cholesterol – are immediately linked to a modern lifestyle characterized by sedentary living, chronic stress, and high intake of energy-dense foods and recreational drugs (Stevens et al., 2009). In addition, following calls from the World Health Organization's (2015/(1946) inclusive conception of health, researchers, civil society, and politicians have been pushing to extend policy goals from preventing and reducing disease towards promoting people's holistic physical, mental, and social well-being (Carlisle and Hanlon, 2008, Hanratty and Farmer, 2012, Huppert and So, 2013, Marks and Shah, 2004, Schulte et al., 2015).

Practically all modern lifestyle health risks (and resulting diseases) are directly affected by people's individual health behaviours — be it physical activity, diet, recreational drug use, medication adherence, or preventive and rehabilitative exercises (Glanz et al., 2008, Schroeder, 2007). By one estimate, three quarters of all health care costs in the US are attributable to chronic diseases caused by poor health behaviours (Woolf, 2008), the effective management of which again requires patients to change their behaviours (Sola et al., 2015). Similarly, research indicates that well-being can be significantly improved through small individual behaviours (Lyubomirsky and Layous, 2013, Seligman, 2011). Behaviour change has therefore become one of the most important and frequently targeted levers for reducing the burden of preventable disease and death and increasing well-being (Glanz, K., Rimer, B. K., & Viswanath, K, 2008, p. xiii).

A main factor driving behaviour change is the individual's motivation. Even if different theories contain different motivational constructs, “the processes that direct and energize behaviour” (Reeve, 2014, p. 8) feature prominently across health behaviour change theories (Glanz and Bishop, 2010, Michie et al., 2011b). Motives are a core target of a wide range of established behaviour change techniques (Michie et al., 2011a,b).

However, following self-determination theory (SDT), a well-established motivation theory, not all forms of motivation are equal (Deci and Ryan, 2012). A crucial consideration is whether behaviour is intrinsically or extrinsically motivated. Intrinsic motivation describes activities done ‘for their own sake,’ which satisfy basic psychological needs for autonomy, competence, and relatedness, giving rise to the experience of volition, willingness, and enjoyment. Extrinsically motivated activity is done for an outcome separable from the activity itself, like rewards or punishments, which thwarts autonomy need satisfaction and gives rise to experiences of unwillingness, tension, and coercion (Deci and Ryan, 2012). In recent years, SDT has become a key framework for health behaviour interventions and studies. A large number of studies have demonstrated advantages of intrinsic over extrinsic motivation with regard to health behaviours (Fortier et al., 2012, Ng et al., 2012, Patrick and Williams, 2012, Teixeira et al., 2012b). Not only is intrinsically motivated behaviour change more sustainable than extrinsically motivated change (Teixeira, Silva, Mata, Palmeira, & Markland, 2012): satisfying the psychological needs that intrinsically motivate behaviour also *directly* contributes to mental and social well-being (Ryan, Huta, & Deci, 2008; Ryan, Patrick, Deci, & William, 2008).

In short, in our modern life world, health and well-being strongly depend on the individual's health behaviours, motivation is a major factor of health behaviour change, and intrinsically motivated behaviour change is desirable as it is both sustained and directly contributes to well-being. This raises the immediate question what kind of interventions are best positioned to intrinsically motivate health behaviour change.

### 1.2 Computing technology for health behaviour change and well-being

The last two decades have seen the rapid ascent of computing technology for health behaviour change and well-being (Glanz, K., Rimer, B. K., & Viswanath, K, 2008, pp. 8–9), with common labels like persuasive technology (Fogg, 2003) or positive computing (Calvo and Peters, 2014). This includes a broad range of consumer applications for monitoring and managing one's own health and well-being (Knight et al., 2015, Martínez-Pérez et al., 2013, Middelweerd et al., 2014), such as the recent slew of “quantified self” (Wolf, 2009) or “personal informatics” tools for collecting and reflecting on information about the self (Li et al., 2010).

One important sector is serious games for health (Wattanasoontorn et al., 2013), games used to drive health-related outcomes. The majority of these are “health behaviour change games” (Baranowski et al., 2008) or “health games” (Kharrazi et al., 2012) affecting the health behaviours of health care receivers (and not e.g. training health care providers) (Wattanasoontorn et al., 2013). Applications and research have mainly targeted physical activity, nutrition, and stroke rehabilitation, with an about equal share of (a) “exergames” or “active video games” directly requiring physical activity as input, (b) behavioural games focusing specific behaviours, (c) rehabilitation games guiding rehabilitative movements, and (d) educational games targeting belief and attitude change as a precondition to behaviour change (Kharrazi et al., 2012). Like serious games in general, health games have seen rapid growth (Kharrazi et al., 2012), with numerous systematic reviews assessing their effectiveness (DeSmet et al., 2014, DeSmet et al., 2015, Gao et al., 2015, LeBlanc et al., 2013, Lu et al., 2013, Papastergiou, 2009, Primack et al., 2012, Theng et al., 2015).

A main rationale for using games for serious purposes like health is their ability to motivate: Games are systems purpose-built for enjoyment and engagement (Deterding, 2015b). Research has confirmed that well-designed games are enjoyable and engaging because playing them provides basic need satisfaction (Mekler et al., 2014, Przybylski et al., 2010, Tamborini et al., 2011). Turning health communication or health behaviour change programs into games might thus be a good way to intrinsically motivate users to expose themselves to and continually engage with these programs (Baranowski et al., 2008; though see Wouters et al., 2013).

However, the broad adoption of health games has faced major hurdles. One is their high cost of production and design complexity: Health games are typically bespoke interventions for a small target health behaviour and population, and game development is a cost- and time-intensive process, especially if one desires to compete with the degree of “polish” of professional, big studio entertainment games. Thus, there is no developed market and business model for health games, wherefore the entertainment game and the health industries have by and large not moved into the space (Parker, n.d, Sawyer, 2014).

A second adoption hurdle is that most health games are delivered through a dedicated device like a game console, and require users to create committed spaces and times in their life for gameplay. This demand often clashes with people's varied access to technology, their daily routines and rituals, as well as busy and constantly shifting schedules (Munson et al., 2015).

### 1.3 Gamification: a new model?

One possible way of overcoming these hurdles is presented by gamification, which is defined as “the use of game design elements in non-game contexts” (Deterding et al., 2011; see Seaborn and Fels, 2015 for a review). The underlying idea of gamification is to use the specific design features or “motivational affordances” (Deterding, 2011, Zhang, 2008) of entertainment games in other systems to make engagement with these more motivating.^1^Appealing to established theories of intrinsic motivation, gamified systems commonly employ motivational features like immediate success feedback, continuous progress feedback, or goal-setting through interface elements like point scores, badges, levels, or challenges and competitions; relatedness support, social feedback, recognition, and comparison through leaderboards, teams, or communication functions; and autonomy support through customizable avatars and environments, user choice in goals and activities, or narratives providing emotional and value-based rationales for an activity (cf. Ryan and Rigby, 2011, Seaborn and Fels, 2015).

Since its emergence around 2010, gamification has seen a groundswell of interest in industry and academia, easily outstripping persuasive technology in publication volume (Hamari, Koivisto, & Pakkanen, 2014). By one estimate, the gamification market is poised to reach 2.8 billion US dollars by 2016 (Meloni and Gruener, 2012). It is little wonder, then, that several scholars have pointed to health gamification as a promising new approach to health behaviour change (Cugelman, 2013, King et al., 2013, Munson et al., 2015, Pereira et al., 2014, Sola et al., 2015). Popular examples are *Nike+*^2^, a system of activity trackers and applications that translate measured physical exertion into so-called “NikeFuel points” which then become enrolled in competitions with friends, the unlocking of achievements, or social sharing; *Zombies*, *Run!*^3^, a mobile application that motivates running through wrapping runs into an audio-delivered story of surviving a Zombie apocalypse; or *SuperBetter*^4^, a web platform that helps people achieve their health goals by building psychological resilience, breaking goals into smaller achievable tasks and wrapping these into layers of narrative and social support.

Conceptually, health gamification sits at the intersection of persuasive technology, serious games, and personal informatics (Cugelman, 2013, Munson et al., 2015): Like persuasive technology, it revolves around the application of specific design principles or features that drive targeted behaviours and experiences. Several authors have in fact suggested that many game design elements can be mapped to established behaviour change techniques (Cheek et al., 2015, Cugelman, 2013, King et al., 2013). Like serious games, gamification aims to drive these behaviours through the intrinsically motivating qualities of well-designed games. Like personal informatics, gamification usually revolves around the tracking of individual behaviours, only that these are then not only displayed to the user, but enrolled in some form of goal-setting and progress feedback. Indeed, many applications commonly classified as gamification are also labelled personal informatics, and gamification is seen as a way to sustain engagement with personal informatics applications (e.g., Morschheuser et al., 2014).

### 1.4 Promises of gamification for health and well-being

The reasons why gamification is potentially relevant to health behaviour change today, and the shortcomings of other digital health and well-being interventions include:

1 *Intrinsic motivation.* Like games, gamified systems can intrinsically motivate the initiation and continued performance of health and well-being behaviours (Deterding, 2015b for similar arguments regarding gamification in general; King et al., 2013, Munson et al., 2015, Pereira et al., 2014; cf. Seaborn and Fels, 2015, Sola et al., 2015). In contrast, personal informatics can lack sustained appeal, and persuasive technologies often employ extrinsic motivators like social pressure or overt rewards (Oinas-Kukkonen and Harjumaa, 2009).
2 *Broad accessibility through mobile technology and ubiquitous sensors.* Activity trackers and mobile phones, equipped with powerful sensing, processing, storage, and display capacities, are excellent and widely available platforms to extend a game layer to everyday health behaviours, making gamified applications potentially more accessible than health games which rely on bespoke gaming devices (King et al., 2013, Lister et al., 2014, Sawyer, 2014).
3 *Broad appeal*. As wider and wider audiences play games, games and game design elements become approachable and appealing to wider populations (King et al., 2013).
4 *Broad applicability*. Current health gamification domains cover all major chronic health risks: physical activity, diet and weight management, medication adherence, rehabilitation, mental well-being, drug use, patient activation around chronic diseases like Diabetes, cancer, or asthma (Munson et al., 2015, Pereira et al., 2014, Sola et al., 2015).
5 *Cost-benefit efficiency*. Retro-fitting existing health systems and enhancing new ones with an engaging “game layer” may be faster, most cost-benefit efficient, and more scalable than the development of full-fledged health games (Munson et al., 2015, Sawyer, 2014).
6 *Everyday life fit*. Gamified systems using mobile phones or activity trackers can encompass practically all trackable everyday activity, unlike health games requiring people to add dedicated time and space to their life (Munson et al., 2015). Whereas standard health games typically try to fit another *additional* activity into people's schedules, gamification aims to *reorganise* already-ongoing everyday conduct in a more well-being conducive manner (Deterding, 2015b; see Hassenzahl and Laschke, 2015).
7 *Supporting well-being*. Beyond motivating health behaviours, engaging with gamified applications can directly contribute to well-being by generating positive experiences of basic psychological need satisfaction as well as other elements of well-being like positive emotions, engagement, relationships, meaning, and accomplishment (cf. Johnson et al., 2013 for a review on well-being effects on video game play; McGonigal, 2011, Pereira et al., 2014).

In short, gamification may realize what games for health doyen Ben Sawyer (2014) dubbed the “new model for health” games should pursue: sensor-based, data-driven, “seductive, ubiquitous, lifelong health interfaces” for well-being self-care.

Promising as gamification for health and well-being may be, the essential question remains whether gamified interventions are effective in driving behaviour change, health, and well-being, and more specifically, whether they manage to do so via intrinsic motivation. These questions are especially relevant as (a) general-purpose literature reviews on gamification have flagged the lack of high-quality effect studies on gamification (Hamari et al., 2014b; cf. Seaborn and Fels, 2015), and (b) critics have objected that gamification often effectively entails standard behavioural reinforcement techniques and reward systems that are extrinsically motivating, not emulating the intrinsically motivating features of well-designed games (Juul, 2011, Walz and Deterding, 2015).

### 1.5 Research goal and questions

To our knowledge, there is no systematic review on the effectiveness and quality of health and well-being gamification applications available. Existing reviews include a survey spanning several application domains which identified four health-related papers (cf. Seaborn and Fels, 2015), a review of gamification features in commercially available health and fitness applications (Lister et al., 2014), a topical review on the use of games, gamification, and virtual environments for diabetes self-management, which identified three studies on gamified applications (Theng et al., 2015), a review focused specifically on the use of (extrinsic) reward systems in health-related gamified applications (Lewis et al., 2016) and a review on the persuasion context of gamified health behaviour support systems (Alahäivälä and Oinas-Kukkonen, 2016). While these reviews offer important and valuable insights, none have examined gamification for both health *and* well-being nor the effectiveness of gamification. Additionally, existing reviews do not directly consider and evaluate the quality of evidence underlying the conclusions drawn. We therefore conducted a systematic literature review of peer-reviewed papers examining the effectiveness of gamified applications for health and well-being, assessing the quality of evidence provided by studies.

We developed four guiding research questions:

• RQ1. What evidence is there for the effectiveness of gamification applied to health and wellbeing?
  o *What is the number and quality of available effect studies?* This follows the observation that gamification research is lacking high-quality effect studies.
  o *What effects are reported?* This follows the question whether health gamification is indeed effective.
• RQ2. How is gamification being applied to health and wellbeing applications?
  o *What game design elements are used and tested?* These questions follow whether health gamification drives outcomes through the same processes of intrinsic motivation that make games engaging, and whether directly supporting well-being through positive experiences.
  o *What delivery platforms are used and tested?* This probes whether current health gamification does make good on the promise of greater accessibility, pervasiveness, and everyday life fit through mobile phones or multiple platforms.
  o *Which theories of motivation (*e.g.*, Self-Determination Theory) are used and tested?* This explores to what extent health gamification explicitly draws on motivational theory and to whether design incorporating these theories leads to better outcomes.
• *RQ3. What audiences are targeted? What effect differences between audiences are observed?* These questions probe whether current applications indeed target a broad range of audiences with equal success. , or whether they only target presumed gaming-affinitive audiences or show less success with non-gaming-affinitive audiences as well as whether.
  o *Is gamification shown to be more effective with gaming affinitive audiences?* This assesses whether the benefits of gamification are limited to audience already familiar with or drawn to game elements as engaging and motivating.
  o *Have the benefits of health gamification been shown to extend to audiences that are not already intrinsically motivated?* This explores whether there is evidence of gamification working when users are not already intrinsically motivated to perform the target activity (e.g., users who voluntarily engage with a fitness app can be assumed to already be intrinsically motivated to exercise).
• *RQ4. What health and well-being domains are targeted?* Beyond a general scoping of the field, this tests whether the claimed broad applicability of gamification indeed holds.

## 2 Methods

The protocol for the review was developed and agreed by the authors prior to commencement. It followed all aspects recommended in the reporting of systematic reviews, namely the PRISMA Checklist and MOOSE Guidelines (Moher et al., 2010). All studies that explored the association between gamification and health were considered for this review. “Gamification” was defined and operationalised as “the use of game design elements in non-game contexts” (Deterding et al., 2011). “Health” and “well-being” were collectively defined and operationalised using the World Health Organization's’s’s’s (1946) inclusive definition of health as “a state of complete physical, mental and social well-being and not merely the absence of disease or infirmity”.

### 2.1 Data collection

The electronic databases in this review were searched on November 19th, 2015 and included those identified as relevant to information technology, social science, psychology and health: Ebscohost (PsychInfo, Medline, CINAHL) (*n* = 33); ProQuest (*n* = 10); Association for Computing Machinery, ACM (*n* = 81); IEEE Xplore (*n* = 36); Web of Science (*n* = 44); Scopus (*n* = 108); Science Direct (*n* = 12) and PubMed (*n* = 39). Three additional studies were identified with a manual search of the reference lists of key studies, including existing gamification reviews, identified during the database search process.

### 2.2 Search terms

Based on prior practice in systematic reviews on gamification and health and well-being (Alahäivälä and Oinas-Kukkonen, 2016, Lewis et al., 2016, Seaborn and Fels, 2015), we used full and truncated search terms capturing gamification, health outcomes, and well-being in the following search string:

Gamif* AND (health OR mental OR anxi* OR depres* OR wellbeing OR well-being).

Mental health related search terms (“mental”, “anxi*” and “depres*”) were added as initial searches failed to capture some expected results.

### 2.3 Inclusion/exclusion criteria

#### 2.3.1 Inclusion criteria

Our review focused on high quality scholarly work reporting original research on the impact and effectiveness of gamification for health and wellbeing. From this focus, we developed the following inclusion criteria:

1 Peer-reviewed (incl. peer-reviewed conference papers)
2 Full papers (incl. full conference papers)
3 Empirical research (qualitative and quantitative)
4 Explained research methods
5 Explicitly state and described gamification as research subject
6 Clearly described gamification elements (type of game design elements)
7 Effect reported in terms of:

a. Impact (affect, behaviour, cognition), and/or
b. User experience — any subjective measure of experience while using the gamified or non-gamified version of the intervention

8 Clearly described outcomes related to health and well-being

Criteria 1–4 were chosen to ensure focus on high-quality work reporting original research. Criteria 3, 4, and 7 were also included to enable assessment of quality of evidence. Criteria 5–6 ensured the paper reported on gamification, not serious games or persuasive technology mislabeled as gamification (a common issue, cf. Seaborn and Fels, 2015). Criteria 7–8 were chosen to assess reported health and well-being outcomes and potential mediators, with user experience included given its prevalence as an outcome measure in gamification research (see Table 1).

**Table 1:** Full paper details and quality of evidence ratings.

Publication	Design	Modality	Domain	Impact	Data analysis	gamification element	Sample size and characteristics	Summary	Rating
Ahtinen et al. (2013)	Single group, month-long field study of ‘Oiva’ tool. Usage acceptance and usefulness of tool measured using interviews and questionnaires. No comparison of gamification to non-gamification.	Mobile phone (android)	Mental health: acceptance and commitment therapy	Behaviour (use of tool) - neutral (no point of comparison). User experience (gamification) — negative effect. Cognition (stress, satisfaction with life) - positive effect. Cognition (psychological flexibility) — no effect.	Qualitative content analysis categorised in 3 themes.	Rewards (virtual roses). Progress (paths).	15: 9 females, Working age.	An ACT (acceptance commitment therapy) — informed mobile app was designed to support learning of wellness skills through ACT-based daily exercises. Progress in the program is presented through various encouraging paths, such as change of color after a number of exercises is completed and a reward of a virtual rose, graphical feedback on progress is given immediately. Although wellness improved, the gamification elements were considered not suitable in the context of wellness and mindfulness. Skepticism towards gamification was expressed by 60%. Rewards were not deemed to sit well with mental wellness and mindfulness.	6.5
Allam et al. (2015)	Random allocation to 1 of 5 conditions (1. control; 2. information section access only; 3. social support only; 4. gamification only; 5. social support & gamification). Outcomes measured using questionnaires.	Website	Physical health: activity, health care utilization, and medication overuse. Mental health: empowerment and knowledge	Behaviour (physical activity, health care utilization) - positive effect of social support & gamification. Cognition (empowerment) — positive effect of social support & gamification. Knowledge (of rheumatoid arthritis) — neutral.	Multilevel linear modeling technique. Time — 3 measurement occasions (1st level), patient (2nd level).	Rewards (points, badges, medals). Leaderboard.	157: Rheumatoid Arthritis patients	Study was designed to look into the effects of a Web-based intervention that included online social support features and gamification on physical activity, health care utilization, medication overuse, empowerment, and Rheumatoid Arthritis (RA) knowledge of RA patients. The effect of gamification on website use was also investigated. A 5-arm parallel randomized controlled trial was conducted. The Web-based intervention had a positive impact (more desirable outcomes) on intervention groups compared to the control group. Social support sections on the website decreased health care utilization and medication overuse and increased empowerment. Gamification alone or with social support increased physical activity and empowerment and decreased health care utilization. Gamified experience increased meaningful website access.	15
Boendermaker et al. (2015)	Two studies: Study 1 — compares four versions of the tool (1. original training, 2. neutral placebo training, 3. gamified, 4. social and gamified).	Study 1 — website. Study 2 — website and mobile	Mental health: Substance use (alcohol)	Study 1.	Repeated measures ANOVAs.	Backstory. Avatar. Social Interaction.	Study 1: 77: 38 females, (18–29 years), Study 2: 64: 39 females, (18–35 years), University students, who regularly drink alcohol.	Study 1 focused on a social and non–social gamified version of an Alcohol/No-Go training, aimed at altering positive associations with alcohol in memory. Study 2 compared a mobile to a stationary computer version of the alcohol approach bias retraining. Results indicate that adding (social) game elements can increase fun and motivation to train using CBM. The social gamified tool improved aspects of the user experience and increased motivation to train. The mobile training appeared to increase motivation to train, but this effect disappeared after controlling for baseline motivation to train.	13
	Study 2 — compares mobile and computer-based interventions.			Cognition (motivation to use tool) — positive effect of social gamified. User experience (ease of use) — gamified less easy to use than non-gamified; gamified easier to use than social gamified. User experience (immersion) — social gamified more immersive than original. User experience (task demand) — gamified more demanding than non-gamified. Behaviour (drinking behaviour) — neutral.					
	Outcomes measured using questionnaires.			Study 2.					
				No relevant differences.					
Cafazzo et al. (2012)	Single group, repeated-measures (prior to using tool cf. while using tool). Outcome is number of times blood glucose readings performed. No comparison of gamification to non-gamification.	Mobile phone (iOS)	Physical health: blood glucose monitoring (diabetes)	Behaviour (blood glucose monitoring) — positive effect. User Experience (satisfaction with tool) — positive. Cognition (self-care, family responsibilities, quality of life) — neutral.	not stated (comparison of means)	Rewards (points). Levels.	20 adolescents (12–16 years)	A 12-week evaluation study of use of a mobile app that aims at increasing the frequency of daily blood glucose measurement. Blood glucose trend analysis was provided with immediate prompting of the participant to suggest both the cause and remedy of the adverse trend. The pilot evaluation showed that the daily average frequency of blood glucose measurement increased 50% (from 2.4 to 3.6 per day, *P* = 0.006, *n* = 12). A total of 161 rewards (average of 8 rewards each) were distributed to participants. Satisfaction was high, with 88% (14/16 participants) stating that they would continue to use the system. Improvements were found in the frequency of blood glucose monitoring in adolescents when using the gamified tool in comparison to not using the gamified tool.	8.5
Chen and Pu (2014)	Comparison of control (no use of tool) with 3 versions of a gamified tool (1. competition, 2. cooperation, 3. hybrid). Outcomes were physical activity (from fitbit), interviews, diary entries and number of messages exchanged. No comparison of gamification to non-gamification.	Mobile phone (android)	Physical health: activity	Behaviour (number of steps) - positive effect of gamified tool (additionally; cooperative and hybrid more steps than competition).	t-tests supplemented with qualitative analysis of diaries and interviews	Rewards (badges, points). Leaderboard.	36: (18 dyads) 17 females, (20–30 years)	Study evaluates HealthyTogether, a mobile game designed to encourage physical activity. Three versions of the game (competition, cooperation, hybrid) were compared in dyads. Participants could send each other messages and earn badges. Users showed a significant increase in physical activity in both the cooperation (by up to 21.1%) and the hybrid setting (by up to 18.2%), but not in the competition setting (by up to 8.8%). In addition the amount of physical activity was found to be correlated with the number of messages sent.	10.5
Dennis and O'Toole (2014)	Between-groups; placebo training (short + long) vs. gamified training conditions (short + long). Outcomes measures via questionnaires.	Mobile (iOS)	Mental health: anxiety/stress	Affect (anxiety and depression) - positive effect of gamified training (greater positive effect with longer compared to shorter gamified training)	ANCOVAs	Rewards (points). Avatar.	38: Long training condition 27 females (mean age 22) 38: Short training condition 28 females (mean age 20 years). Highly trait-anxious adults, psych. Students.	Study examined effects of a gamified Attention-bias modification training (ABMT) mobile application in highly trait-anxious participants. A single session of the active training relative to the placebo training reduced subjective anxiety and observed stress reactivity. The long (45 min), but not the short (25 min) active training condition reduced the core cognitive process implicated in ABMT (threat bias).	10.5
Hall et al., 2013	User evaluation of tool. Usage rates and self-report questionnaires of user experience and wellbeing recorded from users of the tool. No comparison of gamification to non-gamification.	Website (facebook)	Mental health: well-being	Behaviour (answering survey questions) - positive. User experience (rating of tool) - positive.	correlational analysis, analysis method for user experience unstated.	Rewards (points, stars, badges). Social interaction.	121: 37 females	The study evaluates a Gamified Facebook application for the measurement of well-being. A measurement framework for assessing (human) well-being with a much higher observation frequency (e.g. daily) is presented. Gamification provided a suitable environment for exacting accelerated, realistic, truthful self-reporting for the measures of human flourishing (HFS). Higher flourishing scores were correlated with more points, calculation of scores, and charting progress and less correlated with earning badges.	10
Hamari and Koivisto (2015)	Survey measure at a single point of time of users of an existing service. No comparison of gamification and non-gamification.	Mobile (iOS) or Website	Physical health: activity	Behaviour (system use, exercise) — positive. Cognition (intention to recommend) - positive.	non-parametric - component-based PLS (non-parametric alternative to structural equation modeling)	Rewards (Points, and achievements). Levels (level-up system). Social interaction.	200: 102 females, (20–29 years)	Study measured how social influence predicts attitudes, use and further exercise in the context of gamification of exercise. Results show social influence, positive recognition and reciprocity have a positive impact on how much people are willing to exercise as well as their attitudes and willingness to use gamification services. Gamification elements, social influence, positive recognition and reciprocity had a positive impact on participants' desire to exercise. More friends in the game was associated with a larger effect size.	10.5
Jones et al. (2014a)	Alternating treatments design, survey measures taking before and during fruit and vegetable intervention.	Game based rewards provided to heroic characters within a fictional narrative read by teachers	Physical health: nutrition	Behaviour (consumption of fruit and vegetable) - positive.	Conservative Dual Criterion using Monte Carlo simulations to compare fruit and vegetable consumption at different time-points	Rewards (equipment, currency).Narrative. Avatars.	251: 1st–5th grade students	Game based rewards were provided to heroic characters within a fictional narrative read by teachers on days when the school met fruit or vegetable consumption goals. On intervention days, fruit and vegetable consumption increased by 39% and 33% respectively. Teacher surveys indicated that students enjoyed the game and grade 1–3 teachers recommended its use in other schools.	13.5
Jones et al. (2014b)	Alternating treatments design, survey measures taking before and during intervention.	game based rewards provided to heroic characters within a fictional narrative read by teachers	Physical health: nutrition	Behaviour (consumption of fruit and vegetable) - positive.	Conservative Dual Criterion using Monte Carlo simulations to compare time-points for fruit and vegetable consumption. Wilcoxon signed-rank to analyse parent surveys.	Rewards (equipment, currency). Narrative. Avatars.	180: kindergarten – 8th grade students	Game based rewards were provided to heroic characters within a fictional narrative read by teachers on days when the school met fruit or vegetable consumption goals. On intervention days, fruit and vegetable consumption increased by 66% and 44% respectively. In post intervention surveys teachers rated the intervention as practical in the classroom and enjoyed by their students. Parent surveys revealed that children were more willing to try new fruit and vegetable at home and increased their fruit and vegetable consumption following the intervention.	13.5
Kadomura et al. (2014)	Pre-survey, 7 day user test with intervention, post survey. Post intervention interviews. Videos recorded by parents of children using device.	‘Educatableware’ — fork-type device for use with children to improve eating habits	Physical Health: nutrition	Behaviour (teaching children new eating habits) - positive.	Descriptive analysis of surveys, thematic analysis of interviews. Discussion of photos and videos.	Feedback (audio).	5: Children (1–14 years) and parents	Study describes the implementation of the device (a fork that emits a sound when the user is consuming food), and a user test with children. Generally positive results were found in response to the gamified device. Device found to have good usability and the feedback regarding the sounds used was very positive. Three of the five children showed an improvement in food consumption. Additionally, conversation during meal times was reported to improve.	12
Kuramoto et al. (2013)	12-week evaluation of intervention (survey data collected at end of each week of uses). No comparison of gamification and non-gamification. Outcomes measured with questionnaires and sensors in phone).	Mobile device (android)	Physical health: activity (standing on trains)	Behaviour (standing during commute) - positive.	not specified.	Rewards (points). Levels. Avatar.	9 undergrad students	Stand Up, Heroes! (SUH): is a gamified system to motivate commuters to keep standing on crowded public transportation in Japan. In SUH, players have their own avatars which grow larger the longer the player stands. Collecting equipment-item awards increased motivation to stand, however, once all awards were collected, motivation dropped. Watching avatars' growing-up affected participants positively throughout the study. Participants thought the game was fun.	7
Ludden et al. (2014)	Pre- and post-intervention (use of website) evaluation. Survey measures. No comparison of gamification and non-gamification.	Website	Mental health: well-being	Cognition (motivation) - positive. User experience (impression of website) — positive.	Descriptive analysis of survey results. Discussion of interview results.	Challenges. Levels. Progress (map, journey).	13: 10 females, primary school teachers (mean age 38 years)	Study evaluates ‘This Is Your Life’, a training website aimed at personal growth or flourishing. A user-centered design approach was used together with persuasive and gameful design frameworks with primary school teachers. Over half of the participants reported that the design motivated them to do the training; that they would continue using the program; and that they found it challenging and playful.	7
Maher et al. (2015)	RCT with wait-listed control condition. No comparison of gamification to non-gamification. Outcomes measured using questionnaires.	Facebook application	Physical health: activity.	Behaviour (physical activity) — mixed. Cognition (quality of life) - neutral. User experience (engagement) - positive.	Generalized Linear Mixed Models (group: intervention vs control, time: baseline, 8 weeks, and 20 weeks, and group × time interaction entered as fixed effects).	Rewards (achievements, gifts), Leaderboards. Social interaction.	110: teams of 3–8. mean age of 36 years.	Study aimed to determine the efficacy, engagement, and feasibility of a gamified online social networking physical activity intervention with pedometers delivered via Facebook app. Assessments performed at baseline, 8 weeks, and 20 weeks. At 8-week follow-up, intervention participants significantly increased total weekly moderate-vigorous physical activity (MVPA) by 135 min relative to controls (*P* = 0.03). However, statistical differences between groups for total weekly MVPA and walking time were lost at the 20-week follow-up. No significant changes in vigorous physical activity, nor overall quality of life or mental health quality of life at either time point. High levels of engagement with the intervention, and particularly the self-monitoring features, were observed.	12
			Mental health: quality of life						
Reynolds et al. (2013)	Month long intervention with interviews at beginning and end of month. No comparison between gamification and non-gamification.	Wii balance board + Wii Fit Plus software	Physical health: activity	Cognition (motivation to exercise) - positive for beginners, negative for experienced users. User experience (attitude to system) - positive for beginners, negative for experienced users.	Qualitative analysis of interview data.	Rewards (scores, stars). Avatars.	15: 8 females, (18–59 years), beginners (not engaged in regular fitness activity for past year), non-beginners (regularly exercised before starting study)	Study reports a month-long 15-person study of first time Wii Fit users. Participants represent beginners and non-beginners with respect to past fitness experiences and current goals, and these starting points affect their experiences with the system. Beginners respond positively to gamified features. Non-beginners responded negatively (reporting that gamified features slowed down the pace of the exercise; feedback was disliked as praising was considered exaggerated).	6.5
Riva et al. (2014)	RCT (gamified vs non-gamified). Outcomes measured via survey.	Website	Physical health: activity, medication misuse, pain burden.	Cognition (patient empowerment) - positive. Cognition (pain burden) - neutral. Behaviour (medication misuse) - positive. Behaviour (physical exercise) - neutral.	Mixed design ANOVA	Rewards (points). Leaderboard.	51:26 females, (> 18 years), suffering back pain at least 3 months.	Study designed to assess the impact of interactive sections of an Internet-based self-management intervention on patient empowerment, their management of the disease, and health outcomes. Baseline, 4- and 8-week assessments of empowerment, physical exercise, medication misuse, and pain burden. Compared to the control group, the availability of gamified, interactive sections significantly increased patient empowerment and reduced medication misuse in the intervention group. Both the frequency of physical exercise and pain burden decreased, but to equal measures in both groups.	14
			Mental health: empowerment						
Spillers and Asimakopoulos (2014)	Between groups quasi-experimental study (non-gamified social, light gamication and social, heavy gamification and social). Outcomes measured via questionnaires, diary studies, interviews and usage logs.	Mobile (iOS)	Physical health: activity	Behaviour (physical activity) - mixed. Cognition (motivation to exercise) - mixed. User experience (attitude to tools) - mixed.	Not specified	Rewards (badges, prizes). Challenges. Progress. Social Interaction.	15: 7 females, (Age M = 29), experienced iphone app users	Study examines the efficacy of gamification and social elements to improve motivation and lead to short-term positive behaviour change. No clear analysis of the results is undertaken. The majority of results reported are specific “user quotes” but no thematic (or similar) analysis is undertaken and no supported trends in the data are identified by the authors. Running apps designed to track a runner's activity can influence intrinsic motivation regardless of social or gamification elements. Users are more likely to engage in m-health activities if they perceive them as motivating.	7
Thorsteinsen et al. (2014)	RCT. Outcomes measured via surveys and self-reported physical activity. No comparison of gamification to non-gamification.	Website	Physical health: activity	Behaviour (physical activity) - positive. Cognition (motivation) - positive.	ANOVA	Rewards (points). Leaderboards	21: (35–73 years), healthy adults	Study designed to test the effectiveness of a gamified, interactive physical activity intervention. Healthy adults (*n* = 21) (age 35–73) were randomized to the intervention or the control condition. Both groups reported physical activity using daily report forms in four registration weeks during the three-month study: only the experiment condition received access to the intervention. Intervention group reported significantly more physical activity minutes than control group (in week 5 and 9 but not week 12). Participant feedback suggested that gaming components were highly motivating.	8.5
Zuckerman and Gal-Oz (2014)	RCT (3 versions of app). Outcomes measured by log file (movement tracked by phone), questionnaire data and interviews.	Mobile (android)	Physical health: activity	Behaviour (physical activity) - neutral. User Experience (usability) - neutral. User experience (attitude towards system) - mixed.	1) multivariate analysis of variance (3 version) with physical activity as outcome. 2) one-way ANOVA testing the perceived usability of the three StepByStep versions. 3) interview analysis	Rewards (points). Leaderboard.	59: 44 females, (20–27 years), undergrad students	Study evaluates the effectiveness of a gamified application designed to promote routine walking. No differences were found between the gamified and non-gamified versions. The authors speculate that the lack of difference between gamified and non-gamified versions of the tool may be because of the context (physical activity), the timeframe (several days) or the nature of the gamification employed (relatively simple). No differences were found in usability between conditions. Gamification in the form of points was considered meaningless by most users. Attitudes towards leaderboards varied between users (some very interested, some no interest).	11

#### 2.3.2 Exclusion criteria

Our exclusion criteria mirror the focus on high quality scholarly work that reports the impact and effectiveness of gamification for health and well-being. They were particularly framed to exclude duplicate reporting of earlier versions of studies fully reported later. We excluded papers with the following features:

1 Extended abstracts or ‘work-in-progress’ papers
2 Study protocols
3 Covers complete games (serious games) not gamification
4 Gamification is mentioned but not evaluated

Criteria 1–2 exclude peer-reviewed yet early and incomplete versions of studies. Criteria 3–4 exclude studies that mislabel serious games as gamification (see above) or fail to report the concrete intervention in sufficient detail to assess whether it constituted gamification.

### 2.4 Quality assessment tool

We used the quality assessment method presented by Connolly et al. (2012). The tool was explicitly developed to assess the strength of evidence of a total body of work relative to a particular review question. Connolly et al. (2012) used the tool to assess the overall weight of empirical evidence for positive impact and outcomes of games. We applied the tool to our more focused interest in the empirical evidence for the effectiveness of gamification in the health and wellbeing domain. Each final paper included in the review was read and given a score of 1–3 (where 3 denotes high, 2 denotes medium and 1 denotes low on that criterion) across the following five criteria:

1 How appropriate is the research design for addressing the research questions of this review (higher weighting for inclusion of a control group)

a. High — 3 RCT
b. Medium — 2, quasi-experimental controlled study
c. Low — 1, case study, single subject-experimental, pre-test/post-test design

2 How appropriate are the methods and analysis?
3 How generalizable are the findings of this study to the target population with respect to the size and representativeness of the sample? To what extent would the findings be relevant across age groups, gender, ethnicity, etc.
4 How relevant is the particular focus of the study (incl. Conceptual focus, context, sample and measure) for addressing the question of this review?
5 To what extent can the study findings be trusted in answering the study question?

The total weight of evidence for each paper is calculated by adding the scores of all five dimensions, with a range from 5 to 15. Connolly et al.'s (2012 p. 665) analysis of the empirical evidence regarding games and serious games found a mean rating of 8.56 and a mode of 9, which gave us a baseline to evaluate gamification studies against. Connolly et al. (ibid.) found 70 of 129 or 54% of studies to be above the mode, constituting “stronger evidence”. We elected to categorise in slightly more detail, with papers with a rating 8 or below categorised as “weaker evidence”, papers with a rating above 8 to 12 as “moderate evidence”, and papers with a rating above 12 as “stronger evidence”.

### 2.5 Modalities and game design elements

Based on an initial survey, we categorised delivery modalities as mobile (phone), website, social network application, analog, or bespoke device. Given the lack of consensus in the literature regarding definitions and categorizations, game design elements were coded using an adaptation of the systemisation provided by Hamari, Koivisto and Sarsa (2014). Hamari and colleagues identified the following typology: points, leaderboards, achievements/badges, levels, story/theme, clear goals, feedback, rewards, progress and challenge. In the current review, we elected to combine points and badges with other digital rewards (e.g., virtual roses, coins, digital in-app equipment) into a single category labelled ‘rewards’. Additionally, we also coded for the inclusion of an ‘avatar’ or ‘social interaction,’ as these were found to be commonly employed game design elements in the reviewed papers.

### 2.6 Effects

We categorised health and well-being effects as relating to affect (mood), behaviour (i.e., involving real world actions), or cognition (e.g., sense of empowerment, motivation, stress, knowledge of domain). These categories were chosen based on the three-component model of attitudes (Breckler, 1984, Vaughan and Hogg, 1995) with the primary adaptation being the inclusion of knowledge of the target domain as part of the cognition category (knowledge was only assessed in one study (Allam et al., 2015). In addition, multiple studies also assessed user experience (e.g. attitudes towards the gamified intervention itself), which we coded separately. Furthermore, we coded effects as positive, negative, or mixed/neutral, the latter meaning that results were inconclusive or positive for one group and negative for another. If a study assessed health and well-being impacts for multiple dimensions, these were counted separately. For example, a study that finds positive effects on stress and life satisfaction would be counted as two positive impacts on cognition. In contrast, a study that finds a positive impact on life satisfaction for one group of users and negative impact for another would be coded as one neutral/mixed impact on cognition.

### 2.7 Inter-rater reliability

All studies were independently coded by a second reviewer. Inter-rater reliability was determined by the intra-class correlation coefficient (ICC) (Shrout and Fleiss, 1979). This statistic allows for the appropriate calculation of weighted values of rater agreement and accounts for proximity, rather than equality of ratings. A two-way mixed effects, average measures model with absolute agreement was utilized. Independent ratings demonstrated an excellent level of inter-rater reliability (2-way mixed ICC = 0.91; 95% CI 0.77–0.96).

## 3 Results

**Fig. 1:**
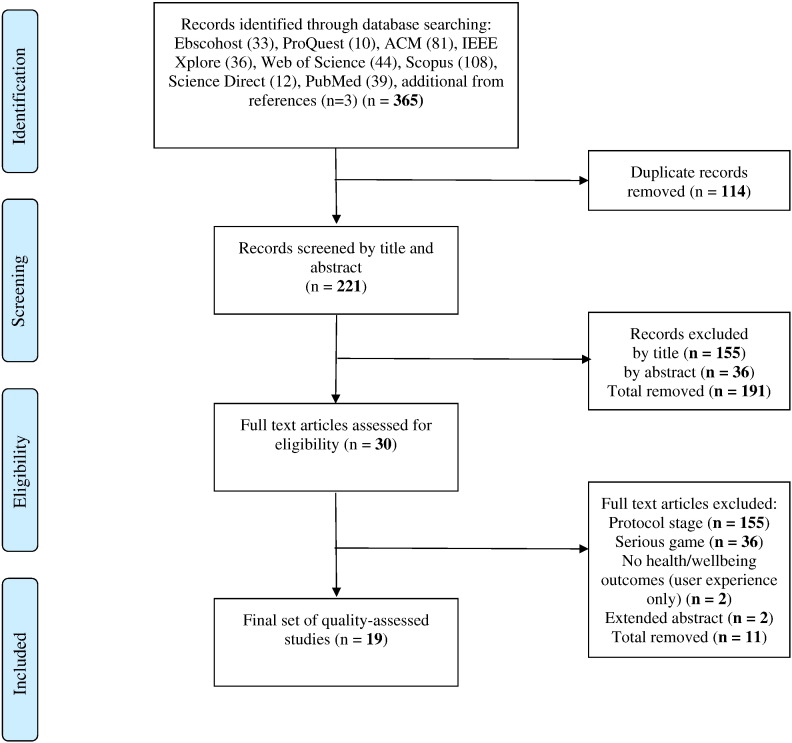
Flow diagram.

Our search identified 365 papers. After removing duplicates 221 papers remained. Of these 191 were removed based on screening of title and abstract. The remaining 30 articles were considered and assessed as full texts. Of them eleven did not pass the inclusion and exclusion criteria. Nineteen final eligible studies remained and were individually assessed for this review. The study selection process is reported as recommended by the PRISMA group (Moher et al., 2010) in Fig. 1.

**Fig. 2:**
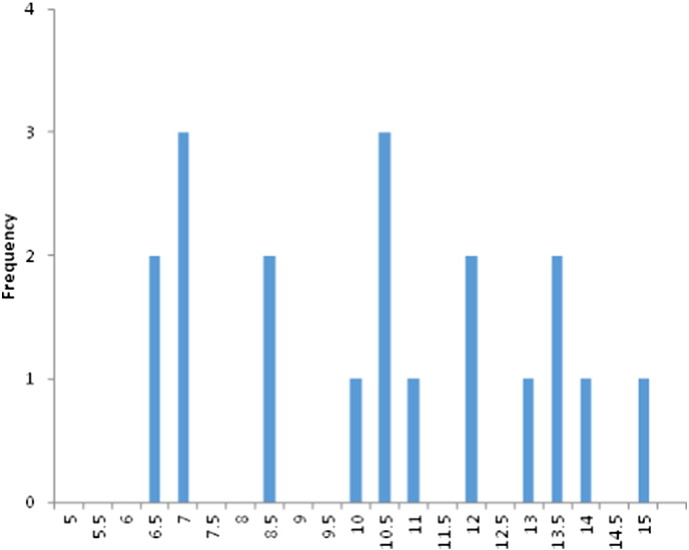
Histogram of quality of evidence ratings.

The final 19 articles eligible for review were then rated for quality of evidence (in relation to the current papers review question, see Table 1). Following Connolly et al. (2012) we calculated the mean (10.3) and mode (10.5) as a means of determining which papers provided relatively weaker or stronger evidence. However, we departed from the approach taken by Connolly and colleagues who assigned papers to two categories (weaker and stronger quality of evidence) and instead categorised papers into three categories (weaker, moderate and stronger evidence). This decision was made as an equal number of papers fell above and below the mode of 10.5 (also the median), which in turn meant that classifying papers with the modal/median score as either weaker or stronger evidence arbitrarily resulted in that category appearing as a majority. Based on this, 8 papers (42%) were categorised as providing weaker evidence, 3 papers (16%) were categorised as providing moderate evidence and 8 papers (42%) were categorised as providing stronger evidence. See Fig. 2 for a histogram displaying quality of evidence ratings.

A closer look into methodologies helps unpack these ratings. The majority (*n* = 11) of studies collected data at multiple timepoints (two or more) from multiple groups or conditions; 6 studies collected data from a single group at multiple timepoints, two from a single group at a single time point. Notably, more than half (*n* = 10) of the studies did not compare gamified and non-gamified versions of the interventions studied. Sample sizes ranged from 5 to 251, sampling methods included both convenient and systematic.

Chief modalities employed were mobile applications (*n* = 7) and websites (*n* = 6), with several studies offering an intervention across both. Two studies each used analog techniques, social networking sites, or bespoke devices, namely a modified fork and a Wii console and Wii Fit board. Game design elements included avatars, challenges, feedback, leaderboards, levels, progress indicators, rewards and story/theme and social interaction (see Table 2). A total of 46 instances of implemented gamification elements were found across the 19 papers. The most commonly employed elements were rewards (*n* = 16), leaderboards (*n* = 6) and avatars (*n* = 6).

**Table 2:** Frequency of user of game design elements.

Game design elements	
Avatars	6
Challenges	2
Feedback	1
Leaderboards	6
Levels	4
Progress	3
Rewards	16
Social interaction	5
Story/theme	3
Total	46

There was a broad variety without discernible patterns in outcome measures (including surveys/questionnaires, interviews, diary entries, videos, log files and equipment readings such as blood glucose readings), target audiences, or contexts, including medical settings, home recovery, self-assessment, health monitoring, stress management, improving eating behaviours, and increasing physical activity.

Overall (see Table 3), positive effects of gamified interventions were reported in the majority of cases (*n* = 22, 59%), with a significant proportion of neutral or mixed effects (*n* = 15, 41%) and no purely negative effects reported. The majority of assessed outcomes were behavioural (*n* = 19, 51%) or cognitive (*n* = 17, 46%). Affect was rarely assessed (*n* = 1, 3%).

**Table 3:** Positive, mixed/neutral and negative health and well-being impacts of gamification.

Impact	Positive	Mixed/neutral	Negative	Number of times each impact assessed
Affect	1			1
Behaviour	13	6		19
Cognition	8	9		17
Number of positive, mixed and negative impacts	22	15	0	37

Beyond health and well-being impacts, 12 studies assessed user experience impacts, with 5 (42%) reporting positive, 5 (42%) reporting mixed and 2 (16%) reporting negative impacts.

## 4 Discussion

For the most part, gamification has been well received; it has been shown to foster positive impacts on affect, behaviour, cognition and user experience. The majority of studies reported gamification had a positive influence on health and well-being. In those cases where gamification had mixed or negative effects, the primary issues seemed to be: 1) the context in which gamification was used (e.g., mindfulness), 2) the manner in which gamification was applied (e.g., exaggerated feedback), or 3) a mismatch between the gamification techniques used and the target audience (e.g., non-beginners feeling that gamification interfered with access to the target activities).

### 4.1 RQ1. What evidence is there for the effectiveness of gamification applied to health and wellbeing?

We assessed evidence based on the number, quality and the reported effects of available studies. We identified a total of 19 studies assessing the effects of gamified health and wellbeing interventions published since 2012 (avg. 5 studies/year). The most comparable serious games for health meta-analysis in terms of inclusion and exclusion criteria is DeSmet et al. (2014), which found 53 studies published between 1989 and 2013 (avg. 2 studies/year). This provides evidence that health gamification research like gamification research in general is progressing at a fast pace (cf. Hamari et al., 2014a,b).

Quality of evidence ratings of existing research conducted by two raters, indicated an equal number of papers were of weak (*n* = 8) or strong (*n* = 8) quality, and the remainder (*n* = 3) were of moderate quality. This suggests that health and wellbeing research is approximately in line with the low evidence quality of gamification research in general (cf. Hamari et al., 2014a, Hamari et al., 2014b) or perhaps slightly better. It is also consistent with the quality of research found in (serious) game research in general: our study found a mean quality rating of 10.3 (with 42% of papers below the mean and classified as providing weaker evidence). In comparison, Connolly et al. (2012 p. 665) reported a mean rating of 8.56 (with 46% of papers classified as providing weaker evidence). While the number of studies included in the current review precludes any firm conclusions, the slightly higher mean quality score found in the current study could indicate the quality of evidence for empirical effectiveness is slightly higher in gamification in health and wellbeing than the broader serious games literature. More broadly, it is worth noting that the small number and low quality ratings of studies included in this review reflect the relative infancy of the gamification field and the formative nature of research conducted to date.

It should also be noted that this analysis of quality of evidence is not intended as a critique of the peer review the selected papers underwent. The papers were categorised as providing lower, moderate or stronger evidence solely with respect to the weight of empirical evidence for health and well-being effects; studies may well be considered differently based on other aims and criteria.

The impact of gamified interventions on health and well-being was found to be predominantly positive (59%). However, a significant portion (41%) of studies reported mixed or neutral effects. More specifically, findings were largely positive for behavioural impacts (13 positive, 6 mixed or neutral), whereas the evidence for cognitive outcomes is less clear-cut, with an approximately equal number of reported positive (*n* = 8) and mixed/neutral (*n* = 9) impacts. Notably, no direct negative impacts on health and wellbeing were reported, although 2 of 12 studies that additionally assessed user experience reported negative impacts on the latter. This picture is more positive than comparable general gamification reviews (cf. Hamari et al., 2014a, Seaborn and Fels, 2015). Current results suggest gamification of health and wellbeing interventions can lead to positive impacts, particularly for behaviours, and is unlikely to produce negative impacts. That being said, gamification should be used with caution when the user experience is critical, e.g. where users can voluntarily opt in and out of the intervention. For example, Spillers and Asimakopoulos (2014) documented user complaints about the poor usability of gamified running apps, which resulted in individual users ceasing to use them. Boendermaker et al. (2015) similarly suggest that gamification may detract from usability and user experience by adding task demands to the interface.

### 4.2 RQ2. How is gamification being applied to health and wellbeing applications?

The majority of papers (*n* = 7) explored mobile devices or websites as the delivery platform (*n* = 6). Positive effects were also found outside the digital domain including a gamified physical display in the classroom (Jones et al., 2014b, Jones et al., 2014a) and a sensor-equipped fork designed to influence children's eating habits (Kadomura et al., 2014). This is in line with the identified promises of *everyday life fit* and *broad accessibility* of gamification through mobile and ubiquitous sensor technology. That being said, there are few studies directly testing the differences and effects of everyday life fit and accessibility in mobile/ubiquitous versus PC/bespoke device-based interventions. Boendermaker et al. (2015) found no difference in effectiveness between a web-based and mobile gamified cognitive bias modification training for alcohol use, but did not explicitly design and control for everyday life fit and accessibility as independent variables.

Although the assessed studies included a broad range of game design elements, there was a clear focus on *rewards*, constituting 16 of a total of 46 instantiations of game design elements across studies (35%), followed by leaderboards and avatars (6 instantiations or 13% each). A notable 84% of all individual studies involved rewards in some form (16 out of 19 studies). Not a single included study captured effects of game design elements on intrinsic motivation as a direct outcome (e.g. motivation to exercise) or mediator for other health and wellbeing outcomes. Taken together with the fact that the majority of studies focused purely behavioural outcomes (see above), this indicates that the dominant theoretical and practical logic of the studied health and wellbeing gamification interventions is positive reinforcement (Deterding, 2015a, pp. 43–45). In other words, the promise of *intrinsically motivating* health behaviour by taking learnings from game design is currently neither explored nor tested.

Eighteen of the 19 included studies implemented *multiple* game elements, and no study tested for the independent effects of individual elements. This makes it difficult to attribute effects clearly to individual game elements, and again underlines the need for more rigorously designed studies. With this caveat, the strongest evidence available does support that rewards^5^ drive health behaviours: Hamari and Koivisto (2015) found rewards in the form of points and achievements to be associated with improvements in desire to exercise. Thorsteinsen et al. (2014) saw points (in combination with leaderboards) to contribute significantly to increased physical activity. Chen and Pu (2014) similarly found that rewards (badges and points) and leaderboards led to an increase in physical activity among dyads working cooperatively (or working in a hybrid cooperative/competitive mode), but not among dyads working competitively. Allam et al. (2015) found that rewards (points, badges and medals in combination with leaderboards) were associated with increased physical activity and sense of empowerment as well as decreased health care utilization among Rheumatoid Arthritis patients. Cafazzo et al. (2012) saw rewards (in the form of points that could be redeemed for prizes) to contribute to the frequency of blood glucose measurement among individuals with type 1 diabetes. Riva et al. (2014) similarly found a positive impact of points (with leaderboards) on outcomes related to chronic back pain, including reduced medication misuse, lowered pain burden, and increased exercise. With a group of highly trait-anxious participants, Dennis and O'Toole (2014) found rewards (in the form of points) associated with reduced subjective anxiety and stress reactivity.

In contrast to these positive outcomes, Maher et al. (2015) report mixed results: rewards (in combination with leaderboards) led to a short-term (8 week follow-up) increase in moderate to vigorous physical activity, but no long-term effects (20 week follow-up). Similarly, they found no impact of gamification on self-reported general or mental quality of life. Studying a mobile application designed to increase routine walking, Zuckerman and Gal-Oz (2014) similarly found no differences between gamified (points and leaderboards) and non-gamified versions. Relatedly, in a qualitative study of gamified mobile running applications, Spillers and Asimakopoulos (2014) observed poor usability of gamified applications leading to users stopping to use them.

Avatars are commonly employed as a gamification technique to represent the user in the application context. Again, the majority of studies found avatars were associated with positive outcomes. Kuramoto et al. (2013) developed an application with an avatar that ‘grew stronger’ the longer users were standing instead of sitting on public transport. They found evidence for increased motivation to stand. Dennis and O'Toole (2014) compared a gamified mobile attention-bias modification training for anxiety using virtual characters with a placebo training and found it to significantly reduce subjective anxiety and stress reactivity. In a series of two studies, Jones et al., 2014a, Jones et al., 2014b found that avatars (in combination with rewards, levels and narrative) led to increased fruit and vegetable consumption among children. Assessing the effectiveness of a gamified (avatar and backstory) application designed to moderate alcohol use, Boendermaker et al. (2015) observed a positive impact on motivation to train; however, participants reported greater task demand associated with the gamified version of the application.

Social Interaction was also commonly employed as a means to engage users and was found to increase user experiences of fun and motivation in the context of moderating alcohol consumption (Boendermaker et al., 2015), to have a positive influence on physical activity (Juho Hamari and Koivisto, 2015, Maher et al., 2015, Spillers and Asimakopoulos, 2014) and flourishing mental health (Hall et al., 2013). Less commonly employed gamed design elements across studies included levels (Cafazzo et al., 2012; Juho Hamari and Koivisto, 2015, Kuramoto et al., 2013, Ludden et al., 2014), progress (Ahtinen et al., 2013, Ludden et al., 2014, Spillers and Asimakopoulos, 2014), story/theme (Boendermaker et al., 2015, Jones et al., 2014b, Jones et al., 2014a), challenges (Ludden et al., 2014, Spillers and Asimakopoulos, 2014) and feedback (Kadomura et al., 2014).

With respect to theories of motivation, very few studies provide insight regarding the extent to which gamification that draws on relevant theory is more effective. Only a minority of studies (*n* = 8) explicitly discuss motivational theory and very few studies (*n* = 3) are conducted in a manner that assesses whether a motivational construct is associated with positive outcomes. Most commonly, self-determination theory and intrinsic/extrinsic motivation were the theories discussed in relation to health gamification (Hall et al., 2013; Juho Hamari and Koivisto, 2015, Riva et al., 2014, Spillers and Asimakopoulos, 2014, Zuckerman and Gal-Oz, 2014). Other theories (relevant to motivation) that were considered include design strategies to reduce attrition and guides for behaviour change (Ahtinen et al., 2013), empowerment (Allam et al., 2015, Riva et al., 2014) and the transtheoretical model of behaviour change (Reynolds et al., 2013).

As discussed above, most studies considered multiple gamification elements simultaneously making it difficult to isolate the effects of individual elements. In some cases, this also makes it more difficult to consider the impact of specific theories of motivation. Hamari and Koivisto (2015) found a positive impact of social norms and recognition providing support for self-determination theory in terms of relatedness of social influence. Similarly, although mixed evidence was found for the impact of the gamification elements used, Zuckerman and Gal-Oz (2014) interpret their results as confirming the value of Nicholson's (2012) concept of ‘meaningful’ gamification and the self-determination driven ideas of informational feedback and customizable elements. Further affirming the notion of ‘meaningful’ gamification, Ahtinen et al. (2013) discuss how their findings highlight the importance of meaningful experiences rather than rewards.

### 4.3 RQ3. What audiences are targeted? What effect differences between audiences are observed?

A broad range of audiences were targeted throughout the research reviewed. While some studies focussed on younger participants (ranging from Kindergarten age (Jones et al., 2014b, Kadomura et al., 2014) to adolescents (Cafazzo et al., 2012), the majority of studies were conducted with adults. Regardless, positive outcomes have been found for children (Jones et al., 2014a, Kadomura et al., 2014), adolescents (Cafazzo et al., 2012) and young adults (Kuramoto et al., 2013, Zuckerman and Gal-Oz, 2014).A small number of studies focussed on specific audiences, such primary school teachers (Ludden et al., 2014), participants with specific health issues like chronic back pain Riva et al., 2014, rheumatoid arthritis (Allam et al., 2015), or high levels of trait anxiety (Dennis and O'Toole, 2014). It is not immediately clear from the reviewed studies what relationship exists between existing gaming affinity or expertise and the effectiveness of gamification as previous experience with digital games is not commonly reported.

Beyond demographics, factors relevant to the potential effectiveness of gamification seem to include the users' personality (Hall et al., 2013), as well as their level of knowledge, expertise, abilities, and basic motivation to engage in the target activity initially. In a study where 15 first-time Wii Fit users were asked to use a Wii balance board to increase their fitness, findings about the effectiveness of gamification were mixed. Only beginners responded positively to gamified elements incorporated into the exercise activities, while these same features had a negative effect on experienced fitness users, leading them to abandon the system as a fitness tool (Reynolds et al., 2013). Non-beginners reported that gamified features slowed down the pace of the exercise, leading to their disengagement, and feedback was disliked, as praising was considered exaggerated.

Importantly, the studies reviewed suggest that the benefits of health gamification extend beyond audiences who have pre-existing motivations to engage in the target activity. Although many (*n* = 11) of the studies involved participants who were likely to have pre-existing motivation, of the studies conducted with participants without existing motivations (*n* = 8), the majority (*n* = 7) showed some positive results. Positive impacts of gamification were found with young children around eating behaviours (Jones et al., 2014a, Jones et al., 2014b, Kadomura et al., 2014); university students regarding alcohol consumption (Boendermaker et al., 2015); commuters with respect to standing Kuramoto et al., 2013 and teachers in relation to positive psychology training. Furthermore, when comparing beginners and experts, Reynolds and colleagues found positive impacts of gamification on exercise behaviour only for the beginners (who are presumably less intrinsically motivated than experts).

### 4.4 RQ4. What health and well-being domains are targeted?

Across fields, the most popular and successful context for the application of gamification is physical health (*n* = 13) and more specifically, its use for motivating individuals to increase their physical activity, or to engage in self-monitoring of fitness levels (*n* = 10). Notably, a positive impact of gamification on physical activity related outcomes are observed in 8 of the 10 studies with mixed effects observed by Maher et al. (2015) and Spillers and Asimakopoulos (2014).

Motivation to exercise is increased largely through “fun” activities, through cooperating, competing, and sharing a common goal with peers or exercise buddies (e.g., Chen and Pu, 2014), or through various other social incentives (e.g., Spillers and Asimakopoulos, 2014). There is evidence that gamification features may be more motivating than exercise alone (Chen and Pu, 2014). Some elements can stimulate increased exercise and reduce physical fatigue (Kuramoto et al., 2013. Gamifying fitness is a way to attract users, encourage participation and motivate behaviour change (Reynolds et al., 2013). There is also evidence to suggest that social influence may play a key role in the influence of gamification on willingness to exercise (Juho Hamari and Koivisto, 2015). While gamified elements can provide motivation to maintain or increase physical activity, such outcomes may not be sustained over time (Thorsteinsen et al., 2014); these responses are not necessarily consistent for all types of users (Reynolds et al., 2013); and not all types of elements help users achieve their fitness goals or positively impact user adoption (Spillers and Asimakopoulos, 2014). Nevertheless, these studies combined lend support to the use of gamification as a viable intervention strategy in fitness contexts. Outside of activity, within the domain of physical health a positive influence of gamification was also found in three studies of nutrition (Jones et al., 2014b, Jones et al., 2014a, Kadomura et al., 2014).

The remaining studies exploring the impact of gamification within the domain of physical health examined illness related issues. Gamification was found to have a positive influence on healthcare utilization (Allam et al., 2015), the reduction of medication misuse (Allam et al., 2015, Riva et al., 2014) and blood glucose monitoring (Cafazzo et al., 2012). In two studies these changes were also associated with a positive influence on patient empowerment (Allam et al., 2015, Riva et al., 2014).

In the domain of mental health, gamification has been shown to have positive effects on wellbeing, personal growth and flourishing (Hall et al., 2013, Ludden et al., 2014) as well as stress and anxiety (Dennis and O'Toole, 2014). This supports the identified promise of gamification to directly *support wellbeing*. More mixed results were found with respect to substance use, with evidence of an increased motivation to train with a gamified version of a tool (designed to alter positive associations with alcohol in memory), alongside evidence of lowered ease of use. However, in a study of mental wellness training, which involved concentration, relaxation and other techniques to encourage changes in thoughts and negative beliefs, gamification was received with skepticism by just over half of the users (Ahtinen et al., 2013). Participants suggested that points, rewards and achievements were a poor fit in the context of mental wellness and mindfulness. However, it is not clear to what extent this point of view is related to the specific types of gamification used in the study and whether the finding would extend to a broader sample.

### 4.5 Limitations

As noted throughout the discussion, the small number and wide variability in the design, quality and health behaviour targets of the gamification studies included in this review limits the conclusions which can be made. There is a need for more well-designed studies comparing gamified and non-gamified interventions: we need randomized controlled trials and double-blind experiments that tease out the effect of individual game design elements on mediators like user experience or motivation and health and wellbeing outcomes, with adequately powered sample sizes, control groups and long-term follow up assessments of outcomes. The studies included in this review typically conflated the assessment of multiple game design elements at once, often involved small sample sizes, did not feature control groups, or only focused on user experience outcomes. Additionally, very few studies have explored the long-term or sustained effects of gamified products, which means that current support for gamification may in part reflect its novelty.

Finally, the heuristic used (positive, negative, neutral) in the current review to evaluate impact, was considered appropriate given the heterogeneity of included studies. However, once more studies on individual gaming elements are completed, future reviews should consider using a more complex heuristic to evaluate impact.

## 5 Conclusions

As the main contributors to health and wellbeing have shifted towards personal health behaviours, policymakers and health care providers are increasingly looking for interventions that motivate positive health behaviour change, particularly interventions leveraging the capabilities of computing technology. Compared to existing approaches like serious games for health or persuasive technology, gamification has been framed as a promising new alternative that embodies a “new model for health”: “seductive, ubiquitous, lifelong health interfaces” for well-being self-care (Sawyer, 2014). More specifically, proponents of gamification for health and wellbeing have highlighted seven potential advantages of gamification: (1) supporting intrinsic motivation (as games have been shown to motivate intrinsically), (2) broad accessibility through mobile technology and ubiquitous sensors, (3) broad appeal across audiences (as gaming has become mainstream), (4) broad applicability across health and wellbeing risks and factors, (5) cost-benefit efficiency of enhancing existing systems (versus building bespoke games), (6) everyday life fit (reorganising existing activity rather than adding additional demands to people's lives), (7) direct wellbeing support (by providing positive experiences).

That being said, little is known whether and how effectively gamification can drive positive health and wellbeing outcomes, let alone deliver on these promises. In response, we conducted a systematic literature review, identifying 19 papers that report empirical evidence on the effect of gamification on health and wellbeing. Just over half (59%) of the studies reported positive effects, whereas 41% reported mixed or neutral effects. This suggests that gamification could have a positive effect on health and wellbeing, especially when applied in a skilled way. The evidence is strongest for the use of gamification to target behavioural outcomes, particularly physical activity, and weakest for its impact on cognitions. There is also initial support for gamification as a tool to support other physical health related outcomes including nutrition and medication use as well as mental health outcomes including wellbeing, personal growth, flourishing, stress and anxiety. However, evidence for the impact of gamification on the user experience, was mixed. Further research that isolates the impacts of gamification (e.g., randomized controlled trials) is needed to determine its effectiveness in the health and wellbeing domain.

In terms of the highlighted promises, little can be said conclusively. No intervention examined intrinsic motivation support (1), as the majority of studies subscribed to a behaviorist reinforcement paradigm. Most studies did employ mobile and/or ubiquitous technology (2), yet no study directly assessed whether they differed in accessibility compared to stationary delivery modes. The range of participant samples employed across studies suggests likely broad appeal across audiences (3) and the wide range of health and wellbeing issues addressed across studies does support broad applicability (4) in principle. None of the studies included assessed cost-benefit efficiency (5) or everyday life fit (6). On a positive note, multiple studies found evidence that gamified interventions did directly support participants' wellbeing (7).

## Footnotes

1 Authors like Deterding et al. (2011) caution to not delimit gamification to a specific design goal like motivation, but grant that motivating behaviours is indeed the overwhelming use case for gamification, as borne out by systematic reviews.

2 https://secure-nikeplus.nike.com/plus/

3 https://zombiesrungame.com

4 http://superbetter.com

5 Because leaderboards were only ever found implemented in conjunction with rewards, we report jointly on both here.
